# Enhancing medication risk communication in developing countries: a cross-sectional survey among doctors and pharmacists in Malaysia

**DOI:** 10.1186/s12889-022-13703-x

**Published:** 2022-07-05

**Authors:** Rema Panickar, Zoriah Aziz, Adeeba Kamarulzaman

**Affiliations:** 1 Faculty of Medicine, University of Malaya, 50603 Kuala Lumpur, Malaysia; 2 National Pharmaceutical Regulatory Agency, Ministry of Health, 46200 Petaling Jaya, Malaysia; 3 Faculty of Pharmacy, MAHSA University, Bandar Saujana Putra, 42610 Jenjarom, Selangor Malaysia

**Keywords:** Safety information, Alerts, Predictors, Survey, Healthcare professionals, Pharmacovigilance, Risk minimization

## Abstract

**Background:**

Medication risk communication is essential to ensure the safe use of medicines. However, very few nations worldwide have established effective risk communication systems. To date, the effectiveness of risk communication among healthcare professionals in Malaysia has never been evaluated. Our study aimed to (i) evaluate doctors’ and pharmacists’ awareness of regulatory risk communication methods; (ii) identify factors predicting the usefulness of these methods; and (iii) compare respondents’ preferences for risk communication to outline suggestions for enhancement.

**Methods:**

We conducted a nationwide cross-sectional survey covering four commonly used risk communications, namely a national drug bulletin, safety alerts, Direct Healthcare Professional Communication letters (DHPCs), and educational materials. Multiple logistic regression analysis was used to determine the association between independent variables and the usefulness of risk communication. We performed qualitative analysis of free-text responses to gain insights on respondents’ perspectives on risk communication.

**Results:**

Of the 1146 responses received, 650 were from pharmacists (56.7%). Among the four methods surveyed, 71.5% of respondents were aware of educational materials, while awareness of the other three methods ranged from 20.7 to 53.9%. Pharmacists had higher awareness of all four methods compared to doctors. Private sector respondents were more aware of DHPCs compared to those from the public sector. The strongest predictors for finding risk communication useful were being a pharmacist [odds ratio (OR) = 18.2; 95% CI: 10.98–30.07; *p* <  0.001], having ≥30 years’ work experience [OR = 4.9; 95% CI: 1.98–12.08; *p* <  0.001], and working in the pharmaceutical industry [OR = 4.6; 95% CI: 1.08–19.72; *p* = 0.039]. Both doctors and pharmacists preferred risk communication in the English-language and electronic format. However, other preferences differed between the professions and sectors. Analysis of free-text comments revealed five core themes to guide risk communication enhancement strategies.

**Conclusions:**

Risk communication awareness differed between public and private sector doctors and pharmacists depending on communication source. Integrating our findings with the theory of effective communication, we provide suggestions for developing strategic plans on enhancing risk communication. Public-private sector collaboration is key in ensuring risk communication effectiveness.

**Supplementary Information:**

The online version contains supplementary material available at 10.1186/s12889-022-13703-x.

## Background

The COVID-19 pandemic has highlighted the importance of effective risk communication in tackling issues such as vaccine-hesitancy and new treatment choices [1, 2]. While medication use is essential, it comes with its own risks and may cause serious harm because of improper use, medication errors, or adverse drug reactions (ADRs) [3]. Accurate, timely, and evidence-based communication to all stakeholders is vital to reduce the risk of ADRs, fear, confusion, and inappropriate medication use [4]. Risk communication which leads to safe and appropriate medication use could have substantial financial impact, considering that global medication spending involves a trillion-dollar industry [5]. However, achievable and sustainable steps to implement effective medication risk communication worldwide remain elusive [6].

Risk communication occurs at every stage of the pharmacovigilance risk management process [7], encompassing the exchange of information and advice on medication safety between experts or regulatory officials and medicinal product users. The ultimate aim is to allow informed therapeutic decisions to be made by healthcare professionals and the public [8, 9]. While communication is a fundamental process of life, communicating the risks of medicinal products is often complicated [10]. Currently, very few countries have proven effective communication strategies in place [6]. The World Health Organization’s (WHO) decade-long efforts to enhance risk communication [11] continue with the latest guidelines on managing an infodemic [1]. WHO emphasizes the need to understand the knowledge, perceptions, beliefs, and practices of all stakeholders [9, 11] while tailoring guidance to encompass the latest advances in communication methods [1].

In Malaysia, medication risk communication is coordinated by the Pharmacovigilance Section of the National Pharmaceutical Regulatory Agency (NPRA). NPRA is the national regulatory authority tasked with ensuring the quality, efficacy and safety of pharmaceutical products in Malaysia. Risk communication is carried out at various levels, for example between regulators and healthcare professionals, industry and the public, or between healthcare professionals and their patients. Currently, most NPRA risk communication targets doctors and pharmacists, who comprise the main handlers of medication [12]. Various risk communication methods have been used in Malaysia over the past 30 years, including press releases, a national bulletin, safety alerts via email or social media, product package inserts, Direct Healthcare Professional Communication (DHPC) letters, and educational materials. However, our previous study [13] revealed that the risk communication methods, specifically related to allopurinol, did not have a significant impact on ADR reporting and prescribing practice.

A recent article [14] highlighted the importance of conducting studies specifically to evaluate the preferences of the target audience in terms of content, form, and delivery of risk communications. While medication risk communication has been carried out in Malaysia for decades, to the best of our knowledge, the effectiveness of this fundamental activity in influencing the knowledge and practice of healthcare professionals has never been established. Factors associated with the usefulness of medication risk communication have also never been identified and analysed.

This study focuses on medication risk communication between regulators and healthcare professionals. We aimed to (i) evaluate the awareness of doctors and pharmacists on four available risk communication methods, (ii) identify factors that predict the usefulness of these methods, and (iii) compare the preferences of respondents on risk communication. We included qualitative analysis to gain insights on respondents’ perspectives on risk communication and offer suggestions for developing a strategic plan for risk communication enhancement. These findings are important to outline specific suggestions for improving risk communication, which could be implemented in all nations with developing risk communication systems.

## Methods

### Study design

We conducted a cross-sectional, multicentre, self-administered web-based survey involving doctors and pharmacists across Malaysia from March to June 2021.

### Study population

The Malaysian healthcare system is divided into two categories: (i) the government-funded public sector which is mainly under the purview of the Ministry of Health (MOH), and (ii) the pay-for-service private sector [15, 16]. The public healthcare sector, estimated to serve approximately 65% of the population, comprises hospitals and health clinics where services are heavily subsidised. Meanwhile, the private sector consists of a network of hospitals, specialist or general practitioner clinics, and retail pharmacies. The types and brands of medicines used in these 2 sectors usually vary because of differences in funding [15].

The study population comprises all registered doctors and pharmacists in the Malaysian public and private healthcare sectors. As of June 2020, there were 71,041 doctors (51,912 serving with the MOH) and 19,341 pharmacists (11,616 serving with the MOH) registered in Malaysia [17].

### Inclusion and exclusion criteria

All fully registered doctors and pharmacists who gave their consent to participate in the online survey were included. Doctors and pharmacists undergoing pre-registration training (house officers and provisionally registered pharmacists) and those not currently practising in Malaysia were excluded.

### Sample size

To achieve a 5% margin of error and confidence interval of 95% [18, 19], the calculated minimum sample size required would be 383 for doctors and 377 for pharmacists. However, we aimed to obtain a larger sample than the calculated number to reduce bias and improve the accuracy of our logistic regression model [20].

### Questionnaire

We adapted the English-language survey questions from the Strengthening Collaboration for Operating Pharmacovigilance in Europe (SCOPE) Joint Action Work Package 6: Healthcare Professional Survey [21], with some modifications based on the literature review. The adapted questionnaire contained six domains with 32 items related to medication risk communication.

Our questionnaire covered four risk communication methods used in Malaysia, namely a national drug bulletin called the Malaysian Adverse Drug Reactions Advisory Committee (MADRAC) bulletin, NPRA safety alerts, DHPCs, and educational materials. Table 1 [22, 23] shows a comparison of these four methods. For each method, respondents were asked to respond on (a) their awareness of the method,^1^ (b) how useful they find the method,^2^ and (c) how likely they would take the action recommended in the communication.^3^

**Table 1:** Characteristics of the four medication risk communication methods included in this study [22, 23]

	Risk communication method			
	MADRAC Bulletin	Safety Alert	DHPC	Educational material
**Author**	NPRA	NPRA	Product registration holder, reviewed by NPRA	Product registration holder, NPRA, MOH
**Target audience**	Healthcare professionals	Healthcare professionals	Healthcare professionals	Healthcare professionals or patients
**Dissemination method**	• Email to healthcare professionals in NPRA mailing list, administrative heads of healthcare facilities, professional associations • NPRA website		By product registration holder directly to healthcare professionals who use the product mentioned in DHPC	• By product registration holder or MOH directly to healthcare professionals • By healthcare professionals to patients
**Content**	• Local ADR case reports • MADRAC activities • Latest pharmacovigilance activities	• Latest or emerging safety issues • Summary of DHPCs	Important changes to medicinal product information.	Additional information to minimize risk of using the product, in addition to package insert information.
**Frequency of publication**	Every four months	As required, depending on emerging safety issues.		

*ADR* Adverse drug reaction; *DHPC* Direct Healthcare Professional Communication letter; *MADRAC* Malaysian Adverse Drug Reactions Advisory Committee; *MOH* Ministry of Health; *NPRA* National Pharmaceutical Regulatory Agency

We assessed the preferences of respondents regarding risk communication and perceptions on the factors that may affect their response to the risk communication through eight questions. The questionnaire included five optional free-text boxes allowing respondents to leave comments on each of the four methods and suggestions for improving risk communication.

For assessment of face and content validity of the questionnaire, we distributed the adapted questionnaire to a group of six experts in medication risk communication or pharmacovigilance. The experts were asked to rate their judgment on the relevance of each item to the measured domain using a 4-point Likert scale. We calculated the content validity index (CVI) (see Additional file 1) to allow objective assessment of content validity, using a CVI cut-off score of at least 0.83 as evaluation was carried out by six experts [24–27]. A scale-level CVI averaging (S-CVI/Avg) score of 0.97 was obtained, and we modified the questionnaire according to comments from the expert panel.

We piloted the questionnaire on a group of 30 doctors and pharmacists among the target population and modified based on feedback obtained. Data from the pilot study were excluded from the analysis.

### Questionnaire distribution

We used convenience sampling as a list of the complete sampling frame with contact details could not be obtained because of data protection issues. Email invitations to participate in this study were sent out via administrative heads of public healthcare facilities for all states in Malaysia, as well as via professional bodies such as the Malaysian Medical Association, Malaysian Pharmacists Society, and Association of Private Hospitals Malaysia. The email explained the purpose of the study and contained a link to the web-based questionnaire, designed using Google Forms.

Informed consent was obtained from respondents on the first page of the questionnaire. The respondents participated voluntarily and were not given any form of remuneration. We disseminated reminder emails at 3 and 8-weeks following the initial questionnaire distribution.

### Data analysis

#### Quantitative analysis

We analysed the data using IBM SPSS version 26 (SPSS Inc., Chicago, Illinois). Categorical data were presented as absolute numbers or percentages, while means and standard deviations (SD) were determined for the continuous numerical variables. We used the Pearson Chi-squared test of independence or independent t-test to compare the demographic data as well as preferences of doctors and pharmacists on risk communication. Level of significance (α) was set at 0.05.

We performed multiple logistic regression analyses to determine the association of seven independent variables (gender, ethnicity, designation, work setting, work experience, strata, and training experience) with the usefulness of risk communication methods in Malaysia. The dependent variable “Overall, do you think NPRA risk communication is useful?” is a dichotomous measure coded 1 = Yes and 0 = No. Univariate logistic regression was performed to identify variables to be included in the model, based on a *p* <  0.25 significance level to ensure identification of variables known to be important [28]. The best fit model was selected using the Hosmer-Lemeshow test and classification table.

#### Qualitative analysis

We collected qualitative data via five optional free-text boxes within the same questionnaire. We analysed responses obtained from free-text comments using manual thematic analysis, according to the six-phase procedure outlined by Braun and Clarke (2019) [29]. Two researchers (RP, ZA) examined the data before independently assigning initial codes. The codes were reviewed and grouped together through concensus among the two members to identify common themes. We then reviewed these themes before generating a thematic map for the entire dataset. Coding and resulting theme generation were verified by the supervisory team.

## Results

### Socio-demographic characteristics

A total of 1146 healthcare professionals completed the survey, comprising 650 pharmacists (56.7%) and 496 doctors (43.3%) [30]. Table 2 shows details of the respondents’ characteristics. Most respondents (66%) were female with a mean age of 37.4 ± 8.3 years. Over 80% (*n* = 922) of the respondents were healthcare professionals practicing in the public sector, and the majority (85.5%) were from urban areas. A significantly higher percentage of pharmacists reported having attended training on medication safety or risk communication compared to doctors (72.9% vs. 50.2%; *p* <  0.001).

**Table 2:** Socio-demographics of respondents (*N* = 1146)

Demographics	Pharmacists (***n*** = 650)	Doctors (***n*** = 496)	***p*** value
	n (%)	n (%)	
**Gender**			
Male	142 (21.8)	248 (50.0)	**< 0.001**
Female	508 (78.2)	248 (50.0)	
**Age (years)**			
Mean ± SD	35.6 ± 6.9	39.8 ± 9.2	**< 0.001**
Range	25 to 69	27 to 75	
**Ethnicity**			
Malay	268 (41.2)	189 (38.1)	**< 0.001**
Chinese	304 (46.8)	152 (30.6)	
Indian	54 (8.3)	141 (28.4)	
Others	24 (3.7)	14 (2.8)	
**Main employment setting**			
**Public sector**			**< 0.001**
Hospital-based	300 (46.2)	264 (53.2)	
Community-based	109 (16.8)	84 (16.9)	
Administrative	113 (17.4)	50 (10.1)	
Others	4 (0.6)	3 (0.6)	
**Private sector**			**< 0.001**
Hospital-based	40 (6.2)	30 (6.0)	
Community-based	52 (8.0)	60 (12.1)	
Pharmaceutical industry	30 (4.6)	2 (0.4)	
Others	2 (0.3)	3 (0.6)	
**Location strata**			
Urban	541 (83.2)	439 (88.5)	**0.012**
Rural	109 (16.8)	57 (11.5)	
**Work experience (years)**			
Mean ± SD	11.1 ± 6.3	14.1 ± 8.5	**< 0.001**
**Attended training on medication safety**			
Yes	474 (72.9)	249 (50.2)	**< 0.001**
No	176 (27.1)	247 (49.8)	
**Preferred methods of keeping medicines knowledge up-to-date**			
Training or courses	543 (83.5)	268 (54.0)	**< 0.001**
Product package inserts or Consumer medication information leaflets	471 (72.5)	223 (45.0)	**< 0.001**
Medicines reference book	371 (57.1)	303 (61.0)	0.172
NPRA website or bulletin	371 (57.1)	82 (16.5)	**< 0.001**
A mobile phone application	347 (53.4)	183 (36.9)	**< 0.001**
Medical journals	263 (40.5)	252 (50.8)	**< 0.001**

Statistically significant *p*-values are shown in bold

*NPRA* National Pharmaceutical Regulatory Agency, *SD* Standard deviation

The preferred methods of keeping medicines’ knowledge up-to-date varied slightly between doctors and pharmacists. Attending training or courses was the top preference for pharmacists (83.5%) while doctors preferred using medicines reference books (61%). Pharmacists expressed a significantly higher preference for the NPRA website or bulletin compared to doctors (57% vs. 16.5%; *p* <  0.001), while more doctors preferred referring to medical journals (50.8% vs. 40.5%; *p* <  0.001).

### Awareness of the four risk communication methods

#### Overall awareness and usefulness

**Fig. 1:**
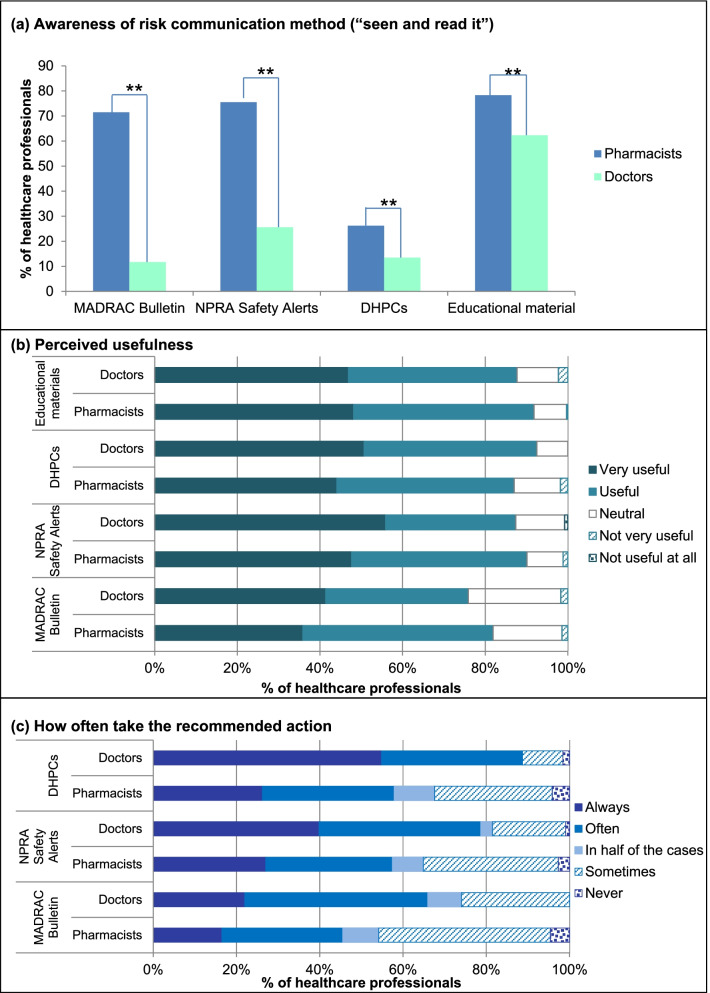
Comparison between doctors and pharmacists on four risk communication methods used in Malaysia. (**a**) Awareness – “have seen and read it”, (**b**) Perceived usefulness, (**c**) How often they take the recommended risk minimization action. Chi-square test, **p < 0.001. DHPCs: Direct Healthcare Professional Communication letters; MADRAC: Malaysian Adverse Drug Reactions Advisory Committee; NPRA: National Pharmaceutical Regulatory Agency

Figure 1 compares respondents’ awareness of the four risk communication methods surveyed, how useful they find each method, and how often they take the recommended risk minimization actions. Overall, 71.5% of respondents had seen and read educational materials [30], while only 20.7% were aware of DHPCs. Figure 1(a) shows that pharmacists had significantly higher awareness of all four methods compared to doctors. Over 70% of pharmacists had seen and read the MADRAC Bulletin and NPRA Safety Alerts, as opposed to less than 26% of doctors. Over 60% of doctors had never heard of the MADRAC Bulletin and DHPCs.

More than 80% of respondents found each of the four methods useful or very useful, with the highest usefulness reported for educational materials (90.2%), as shown in Fig. 1(b). Respondents received educational materials most often from pharmaceutical companies (56.1%), compared to from NPRA (28.2%) or other MOH sources (40.8%).

Figure 1(c) shows that DHPCs were the most likely risk communication method to encourage respondents to take the recommended risk minimization actions, with 67.2% “often” or “always” taking the recommended action, followed by NPRA Safety Alerts (61.9%), and the MADRAC Bulletin (47.9%).

#### Comparison of awareness between public and private sectors

**Fig. 2:**
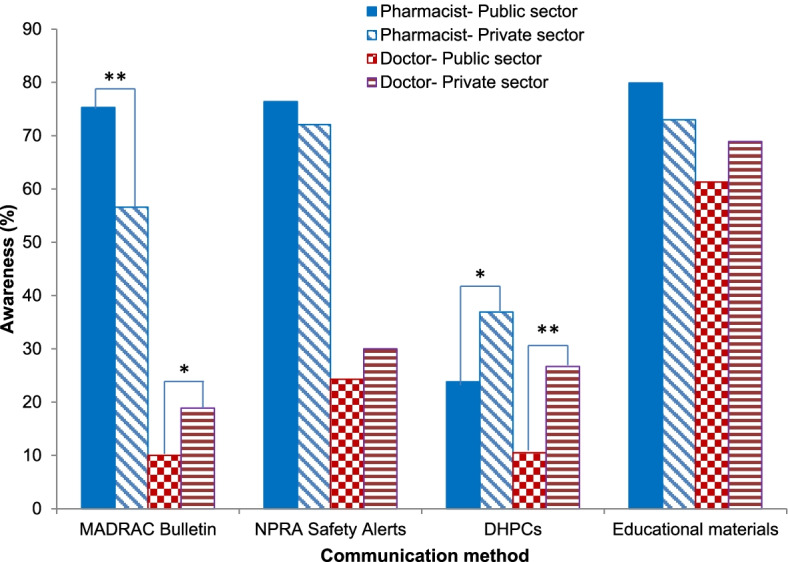
Awareness of public and private sector respondents on four risk communication methods. *p*-values indicate differences between sectors. Chi-square test, *p < 0.05; **p < 0.001. DHPCs: Direct Healthcare Professional Communication letters; MADRAC: Malaysian Adverse Drug Reactions Advisory Committee; NPRA: National Pharmaceutical Regulatory Agency

Figure 2 illustrates that more than 70% of pharmacists practicing in the public healthcare sector were aware of the MADRAC Bulletin, NPRA Safety Alerts and educational materials, while private sector pharmacists were significantly more aware of DHPCs (37.1% vs. 23.6%; *p* = 0.002). Public sector doctors had the lowest awareness of all four communication methods.

### Predictors of risk communication usefulness

Table 3 shows the results of univariate logistic regression analysis and multivariate analysis. The univariate analysis revealed that the independent variables, namely respondents’ gender, ethnicity, designation, employment setting, urban or rural strata, length of work experience, and training experience, were significantly associated with the dependent variable “find risk communication useful”. In the multivariate analysis, except for the variable gender, all the variables remained significantly associated with the dependent variable. The model explained between 32.4% (Cox and Snell R^2^) and 44.3% (Nagelkerke R^2^) of the variance in the dependent variable, and correctly classified 79.2% of cases.

**Table 3:** Predictors of risk communication usefulness (*N* = 1146)

Variables	Univariate regression		Multivariate regression	
	Odds ratio (95% CI)	***p*** value	Adjusted odds ratio (95% CI)	***p*** value
**Gender**				
Male	1		1	
Female	2.14 (1.66, 2.75)	**< 0.001**	1.29 (0.92, 1.78)	0.140
**Age group**				
< 45 years	1			
45 years and above	1.16 (0.82, 1.64)	0.394		
**Ethnicity**				
Chinese	1	< 0.001	1	0.013
Malay	1.30 (0.98, 1.71)	0.065	1.85 (1.28, 2.68)	**0.001**
Indian	0.54 (0.38, 0.76)	**< 0.001**	1.35 (0.87, 2.09)	0.184
Other	1.42 (0.69, 2.93)	0.346	1.44 (0.59, 3.50)	0.425
**Designation**				
Specialist	1	< 0.001	1	< 0.001
General practitioner	1.84 (0.96, 3.55)	0.067	2.69 (1.06, 6.84)	**0.038**
Medical officer	0.92 (0.59, 1.44)	0.703	1.07 (0.64, 1.79)	0.797
Consultant	2.03 (1.10, 3.76)	**0.023**	2.06 (1.07, 3.99)	**0.032**
Pharmacist	13.97 (9.21, 21.17)	**< 0.001**	18.17 (10.98, 30.07)	**< 0.001**
**Employment setting**				
Private- community	1	< 0.001	1	0.013
Private- hospital	1.76 (0.94, 3.30)	0.077	1.96 (0.83, 4.61)	0.124
Public- community	1.30 (0.809, 2.08)	0.281	1.53 (0.73, 3.20)	0.257
Public- hospital	1.14 (0.76, 1.71)	0.537	1.54 (0.80, 2.97)	0.192
Public- administrative	3.18 (1.86, 5.42)	**< 0.001**	3.64 (1.66, 7.99)	**0.001**
Pharma industry	7.80 (2.24, 27.09)	**0.001**	4.61 (1.08, 19.72)	**0.039**
Others	1.61 (0.46, 5.67)	0.456	1.93 (0.43, 8.62)	0.388
**Strata**				
Urban	1		1	
Rural	1.61 (1.12, 2.32)	**0.010**	1.71 (1.08, 2.72)	**0.023**
**Work experience**				
< 5 years	1	0.166	1	< 0.001
5 to 9 years	1.02 (0.67, 1.56)	0.917	1.12 (0.65, 1.91)	0.692
10 to 29 years	1.35 (0.93, 1.98)	0.117	2.63 (1.57, 4.42)	**< 0.001**
30 years and above	1.32 (0.66, 2.66)	0.433	4.89 (1.98, 12.08)	**0.001**
**Attended training**				
No	1		1	
Yes	2.66 (2.07, 3.41)	**< 0.001**	1.60 (1.16, 2.21)	**0.004**
**Preferred language**				
Bahasa Melayu	1			
English	1.11 (0.79, 1.57)	0.545		

Forward LR Multiple Logistic model was applied. Model is fit with Hosmer-Lemeshow test *p* = 0.903, Classification table = 79.2%. Statistically significant *p*-values are shown in bold. *CI* Confidence interval

The strongest predictor of finding risk communication useful was being a pharmacist, compared to being a specialist doctor [odds ratio (OR) = 18.2; 95% CI: 10.98–30.07; *p* <  0.001] [30]. Respondents with over 30 years’ work experience were more likely to find NPRA risk communication useful compared to those with less than 5 years’ experience [OR = 4.9; 95% CI 1.98–12.08; *p* <  0.001]. Meanwhile, doctors and pharmacists working in pharmaceutical industry were almost 5 times more likely to find the risk communication methods useful compared to those based in private sector community settings [OR = 4.6; 95% CI: 1.08–19.72; *p* = 0.039]. Respondents working in public sector administrative departments were more likely to find NPRA risk communication useful compared to those based in private sector community settings [OR = 3.6; 95% CI: 1.66–7.99; *p* = 0.001].

### Preferences on risk communication methods

Table 4 shows the comparison of public and private sector respondents’ preferences on risk communication. A significantly higher percentage of private sector pharmacists (97% vs. 83%; *p* <  0.001) and doctors (94% vs. 83%; *p* = 0.01) prefer the medication risk communication to be in English rather than the Malaysian national language, Bahasa Melayu. Almost all respondents find it useful if the safety message is repeated (96%). Pharmacists had a significantly higher preference for electronic versions of risk communication compared to doctors (73% vs. 61%; *p* <  0.001). However, some groups specifically mentioned a strong preference for hardcopy communications, for example doctors in rural health clinics.

**Table 4:** Preferences of pharmacists and doctors on risk communication (*N* = 1146)

Preferences	Pharmacists, n (%)		Doctors, n (%)		χ^**2**^	***p*** value
	Public sector (***n*** = 526)	Private sector (***n*** = 124)	Public sector (***n*** = 401)	Private sector (***n*** = 95)		
**Language**						
English	438 (83.3)	120 (96.8)	334 (83.3)	89 (93.7)	0.07	0.788
Bahasa Melayu	88 (16.7)	4 (3.2)	67 (16.7)	6 (6.3)		
**Repeated message**						
Yes	508 (96.6)	117 (94.4)	388 (96.8)	92 (96.8)	0.31	0.575
**Format**						
Electronic	383 (72.8)	94 (75.8)	242 (60.3)	60 (63.2)	29.1	**< 0.001**
Hardcopy	58 (11.0)	16 (12.9)	98 (24.4)	15 (15.8)		
No preference	85 (16.2)	14 (11.3)	61 (15.2)	20 (21.1)		
**Preferred frequency**						
Immediate update of individual safety issues	287 (54.6)	69 (55.6)	152 (37.9)	47 (49.5)	**24.2**	**< 0.001**
Weekly update of all safety issues	91 (17.3)	35 (28.2)	71 (17.7)	19 (20.0)	0.28	0.595
Monthly update of all safety issues	250 (47.5)	50 (40.3)	191 (47.6)	43 (45.3)	0.12	0.731
Quarterly update of all safety issues	47 (8.9)	4 (3.2)	75 (18.7)	15 (15.8)	27.7	**< 0.001**
**Preferred source**						
NPRA, Ministry of Health Malaysia	507 (96.4)	115 (92.7)	341 (85.0)	79 (83.2)	41.4	**< 0.001**
Professional body	307 (58.4)	89 (71.8)	264 (65.8)	71 (74.7)	5.33	**0.021**
International regulatory agency	294 (55.9)	68 (54.8)	197 (49.1)	45 (47.4)	5.38	**0.020**
Pharmaceutical companies	236 (44.9)	70 (56.5)	125 (31.2)	44 (46.3)	19.6	**< 0.001**
**Preferred channel**						
E-mail	452 (85.9)	107 (86.3)	295 (73.6)	74 (77.9)	24.6	**< 0.001**
National clinical guidelines	232 (44.1)	40 (32.3)	182 (45.4)	53 (55.8)	3.49	0.062
Website	229 (43.5)	67 (54.0)	156 (38.9)	39 (41.1)	4.45	**0.035**
Product package insert	268 (51.0)	52 (41.9)	101 (25.2)	29 (30.5)	62.5	**< 0.001**
Mobile phone text	182 (34.6)	43 (34.7)	151 (37.7)	41 (43.2)	2.04	0.153
Social media	240 (45.6)	29 (23.4)	125 (31.2)	18 (18.9)	19.3	**< 0.001**
Medical journal	126 (24.0)	26 (21.0)	130 (32.4)	39 (41.1)	15.9	**< 0.001**
Consumer medication information leaflet	176 (33.5)	37 (29.8)	68 (17.0)	15 (15.8)	37.8	**< 0.001**
**Factors which contribute to likelihood of reading safety information**						
information is relevant for daily practice	405 (77.0)	86 (69.4)	297 (74.1)	69 (72.6)	0.46	0.500
trust the sender of the safety information	374 (71.1)	96 (77.4)	254 (63.3)	58 (61.1)	11.5	**0.001**
the document is not too lengthy	346 (65.8)	71 (57.3)	238 (59.4)	55 (57.9)	3.08	0.079
**Factors which contribute to likelihood of taking action in response to safety information**						
the adverse drug reaction is severe or causes irreversible harm	410 (77.9)	93 (75.0)	312 (77.8)	73 (76.8)	0.01	0.924
receive sufficient background information	352 (66.9)	78 (62.9)	257 (64.1)	58 (61.1)	0.87	0.352
recommendations are clear	344 (65.4)	65 (52.4)	249 (62.1)	54 (56.8)	0.40	0.526
information is relevant for daily practice	332 (63.1)	61 (49.2)	237 (59.1)	52 (54.7)	0.56	0.453
information is incorporated in clinical or professional society guidelines	266 (50.6)	45 (36.3)	223 (55.6)	52 (54.7)	6.50	**0.011**

Statistically significant *p*-values are shown in bold. *NPRA* National Pharmaceutical Regulatory Agency

In terms of frequency, pharmacists generally preferred to receive immediate updates of individual safety issues (55%), while doctors preferred monthly updates of all safety issues (47%).

The preferred sources of risk communication (85–96%) were from NPRA and MOH. Private sector pharmacists had a significantly higher preference for receiving risk communication from professional bodies such as the Malaysian Pharmacists Society (72% vs. 58%; *p* = 0.006) and from the pharmaceutical industry (57% vs. 45%, *p* = 0.02). Similarly, a higher percentage of private sector compared to public sector doctors preferred to receive risk communication from the pharmaceutical industry (46% vs. 31%, *p* <  0.001).

The preferred channels to receive risk communication were via email (74–86%), national clinical guidelines (42–47%) and website (39–46%). Pharmacists reported a higher preference for risk communication via product package inserts (49% vs. 26%, *p* <  0.001) and consumer medication information leaflets (33% vs. 17%, *p* <  0.001) compared to doctors. In contrast, a significantly higher percentage of doctors preferred to receive risk communication via medical journals (34% vs. 23%; *p* <  0.001). Looking at the different sectors, public sector pharmacists (46% vs. 23%; *p* <  0.001) and doctors (31% vs. 19%; *p* <  0.001) expressed a significantly higher preference for risk communication via social media.

The survey revealed that respondents would only read the safety information if it was relevant to their daily practice (74–76%), they trusted the sender (63–72%), and the document was not too lengthy (59–64%). They would take action in response to the safety information if the ADR was severe or caused irreversible harm (77–78%), and if they received sufficient background information on the basis for the safety message (64–66%). A higher percentage of doctors compared to pharmacists reported that they would take action if the safety information was incorporated in clinical or professional society guidelines (55% vs. 48%; *p* = 0.011).

### Respondents’ perspectives on enhancing medication risk communication

**Fig. 3:**
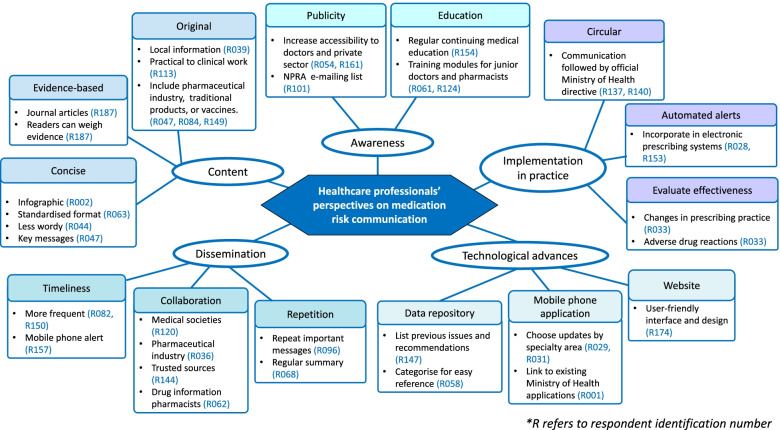
Thematic map of healthcare professionals’ perspectives on medication risk communication derived from the current study

In total, 195 respondents (17%) left 312 free-text comments, the majority of which (*n* = 230; 73.7%) were suggestions for improvement. As shown in Fig. 3, five core themes on risk communication enhancement emerged from analysis of the comments and are supported by illustrative quotations from the participants.

#### Awareness and publicity

The most commonly recurring theme was to increase awareness of the risk communication methods especially among doctors and the private healthcare sector. “Never heard of NPRA, should consider promotion of this service” [respondent 179 (R179), doctor, 38 years]. “Reach out more to community pharmacy or general practitioner settings” (R021, pharmacist, 34 years). “I think the NPRA needs to be more present in platforms commonly accessed by doctors” (R120, doctor, 40 years). Respondents suggested increasing the presence of NPRA in social media. Many respondents were keen for virtual continuing medical education (CME) sessions on medication safety updates to be held regularly.

#### Attractive and concise content

Majority of respondents stated a preference for more concise, evidence-based, and original content. One respondent stated that the MADRAC Bulletin and Safety Alerts contained “mainly cut and paste” information (R030, doctor, 57 years). “Make the risk communication simple and succinct” (R022, pharmacist, 57 years). Many suggested the use of infographics, with a standardized layout to make all risk communication from the national regulatory authority easily recognizable.

#### Dissemination

Respondents preferred more frequent risk communication, with repetition of important messages. “NPRA needs to send same messages repeatedly so that we are aware” (R096, doctor, 52 years). “To distribute educational tools and news more frequently to all public health facilities” (R193, pharmacist, 31 years). Various suggestions were provided to improve the communication outreach, such as collaboration with professional bodies, dissemination through trusted sources including pharmacists or the administrative heads of healthcare departments and sending brief safety alerts via mobile phone with links to further details. “The Malaysian Medical Association has a monthly bulletin. If one page is dedicated for it every month, it would be useful and informative” (R023, doctor, 35 years).

#### Technological advances

Many respondents were keen for a mobile phone application to receive medication risk communication and suggested an “app with automatic messages on drug safety that can be tailored according to specialty area” (R029, doctor, 37 years). Some specific comments were also received regarding improvements required on the regulatory agency website. A number of respondents mentioned the creation of a repository of all previous medication risk communications as a reference source. “It would be great if NPRA could have an electronic repository, allowing healthcare professionals and patients to download safety information as required.” (R036, medical officer in the pharmaceutical industry, 36 years). “Recommend having a categorical listing of important updates and previous updates so that previous information which may have been missed over the years, remain relevant” (R058, pharmacist, 31 years).

#### Implementation of risk minimization measures

Several respondents left comments regarding the actual implementation of risk minimization measures or advice mentioned in the risk communication. “With electronic hospital information systems, good to incorporate automated safety alerts to warn prescribers at point of care” (R028, doctor, 39 years). “Ensure the risk communication is incorporated with changes in practice via circular to both doctors and pharmacists” (R080, pharmacist, 34 years). “Communication between Ministry of Health superiors and the implementing level must be clear and accurate” (R137, doctor, 46 years).

## Discussion

This study aimed to assess the awareness of risk communication among doctors and pharmacists, identify factors which predict the usefulness of risk communication, and determine the methods they preferred for risk communication. The findings reveal that awareness of most risk communication methods is low. Six significant variables were identified that predict those who would find risk communication useful. These are ethnicity, job designation, employment setting, rural or urban areas, length of work experience, and training experience. Analysis of the survey responses revealed five core themes on enhancing risk communication.

Of the four available risk communication methods assessed, doctors and pharmacists had the highest awareness of educational materials. This is probably because these materials are often made available through multiple sources for a prolonged period [23], resulting in increased awareness. About half of the respondents were aware of the MADRAC Bulletin and NPRA Safety Alerts which are directly distributed by the national regulatory authority, most of those aware being pharmacists from the public healthcare sector. Awareness was lowest for DHPCs, which are solely distributed by pharmaceutical companies to users of the medicinal product mentioned in the letter. Our findings indicate that the methods of risk communication dissemination impact the awareness levels.

Almost 60% of respondents had never heard of DHPCs, although these communications have been widely used worldwide since 2012 [14]. However, compared to healthcare professionals from the public sector, those from the private sector had a higher level of awareness of DHPCs. This is possibly because those from the private sector are more often directly involved in purchasing medication from the pharmaceutical companies. Internationally, studies have shown DHPCs to be ineffective due to several factors. DHPCs have been viewed as a defensive practice to transfer responsibility from manufacturers to prescribers, or a form of advertising as it is signed and distributed by the pharmaceutical companies [14]. Surprisingly, our study found that DHPCs were the most effective risk communication method in prompting respondents to take the recommended risk minimization actions. An earlier study also showed DHPCs to be more effective than drug bulletins [31]. Thus, changes to the DHPC dissemination methods are needed to fully utilize this promising communication channel. Besides distribution by the pharmaceutical companies, the DHPCs could be made accessible online through regulatory authority websites to ensure coverage of both public and private sectors. A recent change made in Denmark could be adapted by other countries, whereby DHPCs are still signed by the industry but are disseminated by the Danish Medicines Agency to increase levels of trust [14].

The national regulatory authority, Ministry of Health, and professional bodies emerged as the preferred sources of risk communication in this study, ahead of international regulatory agencies. This concurs with a previous study which reported that Asian health officials preferred guidance from in-country sources [32]. Trust in the source is a major factor in the success of risk communication, with low trust resulting in decreased uptake [33]. When organizations are not transparent in communicating risks, their credibility is reduced [34].

Our study revealed important differences between the responses of doctors and pharmacists, which could be explained by their different professional roles. Pharmacists are much more likely to find risk communication useful, as their role focuses on medications, whereas doctors often cover a range of areas within their professional focus. Our study also showed that pharmacists had a significantly higher preference compared to doctors for risk communication via product package inserts. This is not surprising as most doctors, especially from the public healthcare sector, are not directly involved in handling or dispensing medication [35]. We found that pharmacists are significantly more aware of medication risk communication compared to doctors, most likely because the Malaysian regulatory authority is part of the Ministry of Health Pharmacy Services Programme. A previous study on awareness of medication error reporting also revealed higher awareness among pharmacists [18]. Pharmacists are a trusted source of medication safety information for other healthcare professionals, patients, and the public [12, 36]. As pharmacists are ideally placed across the country in various healthcare sectors, they can play a key role in increasing risk communication outreach to urban and rural areas, other healthcare professionals, policymakers, the pharmaceutical industry, and the general community. Regulatory authorities should conduct regular training for pharmacists on risk communication methods, latest safety issues and effective dissemination. Eventually, these trained pharmacists could serve as the trainers in their respective facilities.

Respondents’ preferences on risk communication as reported in this study are in agreement with the seven key aspects of effective communication, namely a trusted sender, relevant context and content, clear and concise wording, repetition of the message, use of multiple channels to reach target recipients, and suitable for capability of the audience [37, 38]. Therefore, strategies to enhance risk communication should be planned based on these preferences.

Some key suggestions to enhance risk communication are as follows: (1) use multiple sources for dissemination; (2) produce concise and attractive content involving multiple stakeholders; (3) repeat important or urgent messages; (4) increase awareness of regulatory risk communication; (5) enhance existing channels of dissemination; (6) plan technological advances; and (7) improve training for both healthcare professionals and communicators.

Overall, our findings point towards public-private sector collaboration as the key to enhancing risk communication. The same message should be disseminated simultaneously by the national regulatory authority, pharmaceutical companies, and professional bodies to improve outreach as well as overcome trust issues. Further cascading of information by healthcare professionals in administrative departments and pharmacists from all sectors would increase effectiveness of risk communication [39]. Collaboration on technological developments such as mobile phone applications or websites for medication safety updates could also result in more effective systems than those created in silos. Ideally, important safety changes from the risk communication should be integrated into clinical practice guidelines and point-of-care alerts to increase uptake [14].

Medication risk communication in Malaysia has evolved over the past 20 years [13]. Initially producing printed material solely targeting healthcare professionals, NPRA now utilizes electronic communication as well as social media, and disseminates information to consumers [13]. The quality of risk communication content may be enhanced through multidisciplinary collaboration, by inviting non-regulatory healthcare professionals and in the future, consumer group representatives or patients to contribute articles and commentaries. Involvement of different stakeholders with various perspectives would result in communication which appeals to a broader audience [10].

Numerous respondents expressed a preference for risk communication in the format of concise infographics and using simple language. This finding is backed by a recent study emphasizing the use of plain language to get the message across in a transparent, actionable manner [40]. The amended risk communication format should be guided by design science, gathering input from users to make adjustments until the design is finalized [10]. This process would ensure the development of more effective communication tools.

One hurdle faced by regulatory authorities in risk communication is striking a balance between caution and timeliness [6]. Several respondents commented that the NPRA risk communication was delayed compared to the European Medicines Agency or the United States Food and Drug Administration. This is a problem faced by smaller regulatory authorities worldwide [6]. There is a need to develop a risk communication review and approval process which is precise yet prompt [41]. Regulatory authorities do need to practice caution to a certain extent, as the risk communications may elicit variable responses. This has been shown in numerous previous cases, where regulators have been accused of delayed action, secrecy, inaccurate benefit-risk assessment, and external commercial influence [42, 43].

It is encouraging to note that respondents who have received training in medication safety had a significantly higher awareness of NPRA risk communication. However, this survey revealed that only half the doctors and 73% of pharmacists surveyed had received such training. This should be made an essential area of focus. Traditionally, NPRA conducts training on pharmacovigilance and medication safety via physical lectures and workshops [44]. However, the outreach of these methods is poor, and timing may be inconvenient for busy clinicians. The COVID-19 pandemic has accelerated the development of online tools for training, increasing familiarity and acceptance of virtual platforms to improve communication [45]. A recent study showed that a virtual boot camp had a significant impact on improving the knowledge and awareness of healthcare professionals [46]. As a number of our respondents suggested, regular virtual training programs should be conducted with flexible timing to accommodate the schedule of doctors. Effective training would increase awareness of risk communication as well as provide clinicians with the latest knowledge to ensure optimum patient safety.

The importance of training for risk communicators has also been highlighted in previous studies, that showed personnel trained in communications are key to risk communication success and should be given leadership roles [47]. These study findings highlight the need for intensive training on risk assessment and communication to take pharmacovigilance to the next level.

Besides the suggestions mentioned above, evaluation should be conducted for every risk communication effort implemented [48]. Audience feedback should be analyzed to determine further measures for improvement, as effective risk communication is a two-way process [48, 49].

Undeniably, several stumbling blocks exist in the path towards enhancing risk communication in many countries [6, 8], such as budget constraints for the development of communication technology systems and lack of highly trained personnel. Considering these issues, immediate suggestions from these findings emphasize the use of existing platforms to save cost, increase awareness, and reduce information overload for healthcare professionals, while long-term plans should involve technological advances.

### Limitations

This study has several limitations. First, the use of non-probability sampling is not ideal but was unavoidable, as a list of complete sampling frame could not be obtained because of data protection issues. However, this limitation was minimized by obtaining a large sample size and responses from all states in Malaysia, including both urban and rural areas. The responses from doctors were lower than pharmacists, though their number is three times larger than pharmacists. While this was expected based on doctor response rates in other studies, it may increase the chances that data from doctors who responded are not representative of the population. Second, the use of an online survey platform may have excluded non-technologically savvy groups, such as those in areas without internet connection. Third, the entire survey was conducted while the COVID-19 pandemic was raging in Malaysia, therefore there may be a bias in terms of the groups who were likely to participate as many clinicians would have been very occupied with the pandemic response. Finally, our study is subject to the known biases of cross-sectional, self-reporting survey methodology including recall bias, answering tendencies, and misunderstanding on terminology. However, we reduced this limitation by validating the questionnaire with experts and piloting it among the target population. We also added qualitative data analysis to provide a deeper understanding of the respondents’ preferences on risk communication. We acknowledge that our results lack the high level of integration between quantitative and qualitative findings which is the preferred practice for mixed methods research [50, 51]. Nevertheless, our qualitative analysis offers valuable insights which we believe strengthen the study. These insights will help guide the development of a strategic plan for enhancing medication risk communication.

### Future research

Effective risk communication is a multi-stage process. The initial steps are for the message to be sent and received, as have been evaluated in this study. This paper could be used to reach out and collaborate with other stakeholders. The constructive comments and suggestions provided by the respondents have been categorized into different target areas. These could serve as a guide for focus group discussions involving all stakeholders to develop a specific, evidence-based, achievable and sustainable national strategic plan for the enhancement of risk communication.

Moving forward, we need to ensure the message is understood and prompts action or change in behaviour. Evaluation of risk communication effectiveness should be incorporated into the governance structure of regulatory agencies to ensure accountability [6]. National regulatory authorities could collaborate with the pharmaceutical industry and academia to conduct further studies evaluating the impact of risk communication on prescribing practice, ADR reporting rates, and health outcomes.

Following efforts to enhance communication between the regulatory authority and healthcare professionals, robust research must be conducted on communication with patients. Studies on doctor-patient and pharmacist-patient communication should be carried out to determine the views and preferences of both parties. This would reveal if patients of a particular country are prepared to move towards the “partnership” model which is used in Western countries and has been adapted in many Asian countries [52–54].

## Conclusions

Awareness of medication risk communication needs to be increased, especially among doctors and private sector healthcare professionals. Both doctors and pharmacists should play a key role in disseminating risk communication to other healthcare professionals as well as patients. Public-private sector collaboration must be enhanced to increase risk communication outreach and technological developments. Given that effective risk communication is vital for medication safety, our findings have the potential to guide regulatory authorities towards developing national strategic plans for enhancing risk communication.

## Supplementary Information

**Additional file 1.** Content Validity Index calculation.

## Data Availability

The analysis results generated in the current study are presented in this published article. The research datasets will not be publicly available because they contain sensitive material, identifying participant information. The datasets generated for this study are available from the corresponding author on reasonable request.

## Abbreviations

ADR: Adverse drug reaction
CVI: Content validity index
DHPC: Direct Healthcare Professional Communication
MADRAC: Malaysian Adverse Drug Reactions Advisory Committee
MOH: Ministry of Health
NPRA: National Pharmaceutical Regulatory Agency
WHO: World Health Organization

## Footnotes

1 Four answer options were given [1 = Yes, I have seen and read it, 2 = Yes, I have seen but have never read it, 3 = No, I have heard of but never seen it, 4 = No, I have never heard of it].

2 A 5-point Likert scale was used (1 = not useful at all, 2 = not very useful, 3 = neutral, 4 = useful, 5 = very useful).

3 A 5-point Likert scale was used (1 = never, 2 = sometimes, 3 = in half of the cases, 4 = often, 5 = always).
