# Supplementary material for: Enhancing medication risk communication in developing countries: a cross-sectional survey among doctors and pharmacists in Malaysia
Source: BMC Public Health. 2022 Jul 5;22:1293. doi: 10.1186/s12889-022-13703-x (PMC9253255; doi:10.1186/s12889-022-13703-x)
Supplement: Supplementary file 1 — Additional file 1. Content Validity Index calculation. [file 12889_2022_13703_MOESM1_ESM.docx]

**Enhancing medication risk communication in developing countries: a cross-sectional survey among doctors and pharmacists in Malaysia**

**Additional file 1: Content validity index calculation**

**Supplementary Table 1:** Ratings on a 33-item questionnaire by 6 experts for evaluation of content validity

| **Question number** | **Rating** | | | | | |
| --- | --- | --- | --- | --- | --- | --- |
|  | **Expert 1** | **Expert 2** | **Expert 3** | **Expert 4** | **Expert 5** | **Expert 6** |
| **1** | 4 | 4 | 4 | 4 | 4 | 4 |
| **2** | 4 | 4 | 4 | 4 | 4 | 4 |
| **3** | 3 | 4 | 3 | 4 | 4 | 4 |
| **4** | 4 | 4 | 4 | 4 | 4 | 4 |
| **5** | 4 | 3 | 3 | 4 | 4 | 4 |
| **6** | 4 | 3 | 4 | 4 | 4 | 3 |
| **7** | 4 | 4 | 3 | 4 | 4 | 4 |
| **8** | 4 | 4 | 4 | 4 | 3 | 3 |
| **9** | 3 | 4 | 3 | 3 | 4 | 3 |
| **10** | 4 | 4 | 3 | 4 | 4 | 4 |
| **11** | 3 | 4 | 4 | 3 | 4 | 4 |
| **12** | 3 | 4 | 4 | 3 | 4 | 3 |
| **13** | 4 | 4 | 4 | 4 | 4 | 4 |
| **14** | 3 | 4 | 4 | 3 | 4 | 4 |
| **15** | 3 | 4 | 4 | 3 | 4 | 3 |
| **16** | 4 | 4 | 4 | 4 | 4 | 4 |
| **17** | 3 | 4 | 4 | 3 | 4 | 4 |
| **18** | 3 | 4 | 4 | 3 | 4 | 3 |
| **19** | 2 | 4 | 4 | 1 | 2 | 4 |
| **20** | 3 | 4 | 4 | 4 | 4 | 4 |
| **21** | 3 | 4 | 4 | 3 | 4 | 4 |
| **22** | 3 | 4 | 4 | 4 | 4 | 4 |
| **23** | 4 | 4 | 4 | 4 | 4 | 4 |
| **24** | 4 | 3 | 3 | 4 | 3 | 4 |
| **25** | 3 | 4 | 4 | 4 | 4 | 4 |
| **26** | 4 | 4 | 4 | 4 | 4 | 4 |
| **27** | 4 | 3 | 4 | 4 | 4 | 4 |
| **28** | 4 | 4 | 4 | 4 | 4 | 3 |
| **29** | 3 | 4 | 4 | 4 | 4 | 2 |
| **30** | 4 | 4 | 4 | 4 | 4 | 4 |
| **31** | 4 | 4 | 4 | 4 | 4 | 4 |
| **32** | 4 | 4 | 2 | 4 | 4 | 4 |
| **33** | 4 | 4 | 3 | 4 | 4 | 4 |

Rating scale (Degree of relevance):

1 = the item is not relevant to the measured domain

2 = the item is somewhat relevant to the measured domain

3 = the item is relevant to the measured domain

4 = the item is highly relevant to the measured domain

**Supplementary Table 2:** Content Validity Index (CVI) calculation

| **Question number** | **Recoded rating** | | | | | |  |  |  |  |
| --- | --- | --- | --- | --- | --- | --- | --- | --- | --- | --- |
|  | **Expert 1** | **Expert 2** | **Expert 3** | **Expert 4** | **Expert 5** | **Expert 6** |  | **Experts in agreement** | **I-CVI** | **UA** |
| 1 | 1 | 1 | 1 | 1 | 1 | 1 |  | 6 | 1.00 | 1 |
| 2 | 1 | 1 | 1 | 1 | 1 | 1 |  | 6 | 1.00 | 1 |
| 3 | 1 | 1 | 1 | 1 | 1 | 1 |  | 6 | 1.00 | 1 |
| 4 | 1 | 1 | 1 | 1 | 1 | 1 |  | 6 | 1.00 | 1 |
| 5 | 1 | 1 | 1 | 1 | 1 | 1 |  | 6 | 1.00 | 1 |
| 6 | 1 | 1 | 1 | 1 | 1 | 1 |  | 6 | 1.00 | 1 |
| 7 | 1 | 1 | 1 | 1 | 1 | 1 |  | 6 | 1.00 | 1 |
| 8 | 1 | 1 | 1 | 1 | 1 | 1 |  | 6 | 1.00 | 1 |
| 9 | 1 | 1 | 1 | 1 | 1 | 1 |  | 6 | 1.00 | 1 |
| 10 | 1 | 1 | 1 | 1 | 1 | 1 |  | 6 | 1.00 | 1 |
| 11 | 1 | 1 | 1 | 1 | 1 | 1 |  | 6 | 1.00 | 1 |
| 12 | 1 | 1 | 1 | 1 | 1 | 1 |  | 6 | 1.00 | 1 |
| 13 | 1 | 1 | 1 | 1 | 1 | 1 |  | 6 | 1.00 | 1 |
| 14 | 1 | 1 | 1 | 1 | 1 | 1 |  | 6 | 1.00 | 1 |
| 15 | 1 | 1 | 1 | 1 | 1 | 1 |  | 6 | 1.00 | 1 |
| 16 | 1 | 1 | 1 | 1 | 1 | 1 |  | 6 | 1.00 | 1 |
| 17 | 1 | 1 | 1 | 1 | 1 | 1 |  | 6 | 1.00 | 1 |
| 18 | 1 | 1 | 1 | 1 | 1 | 1 |  | 6 | 1.00 | 1 |
| 19 | 0 | 1 | 1 | 0 | 0 | 1 |  | 3 | 0.50^a^ | 0 |
| 20 | 1 | 1 | 1 | 1 | 1 | 1 |  | 6 | 1.00 | 1 |
| 21 | 1 | 1 | 1 | 1 | 1 | 1 |  | 6 | 1.00 | 1 |
| 22 | 1 | 1 | 1 | 1 | 1 | 1 |  | 6 | 1.00 | 1 |
| 23 | 1 | 1 | 1 | 1 | 1 | 1 |  | 6 | 1.00 | 1 |
| 24 | 1 | 1 | 1 | 1 | 1 | 1 |  | 6 | 1.00 | 1 |
| 25 | 1 | 1 | 1 | 1 | 1 | 1 |  | 6 | 1.00 | 1 |
| 26 | 1 | 1 | 1 | 1 | 1 | 1 |  | 6 | 1.00 | 1 |
| 27 | 1 | 1 | 1 | 1 | 1 | 1 |  | 6 | 1.00 | 1 |
| 28 | 1 | 1 | 1 | 1 | 1 | 1 |  | 6 | 1.00 | 1 |
| 29 | 1 | 1 | 1 | 1 | 1 | 0 |  | 5 | 0.83 | 0 |
| 30 | 1 | 1 | 1 | 1 | 1 | 1 |  | 6 | 1.00 | 1 |
| 31 | 1 | 1 | 1 | 1 | 1 | 1 |  | 6 | 1.00 | 1 |
| 32 | 1 | 1 | 0 | 1 | 1 | 1 |  | 5 | 0.83 | 0 |
| 33 | 1 | 1 | 1 | 1 | 1 | 1 |  | 6 | 1.00 | 1 |
|  |  |  |  |  |  |  |  | **S-CVI/Averaging** | 0.97 |  |
| **Proportion relevance** | 0.97 | 1.00 | 0.97 | 0.97 | 0.97 | 0.97 |  | **S-CVI/UA** |  | 0.91 |
|  | **Average proportion of items judged as relevant across the six experts** | | | | | | 0.97 |  |  |  |

Recoding of rating scale from Supp. Table 1:

0 = not relevant (rating 1 or 2)

1 = relevant (rating 3 or 4)

^a^Question 19 scored below the cut-off point of 0.83. However, this question was included in the final questionnaire as it was part of the original adapted questionnaire and deemed important by supervisors. I-CVI: item-level content validity index; UA: universal agreement; S-CVI: scale-level content validity index
